# Definition, assessment and treatment of cognitive impairment associated with schizophrenia: expert opinion and practical recommendations

**DOI:** 10.3389/fpsyt.2024.1451832

**Published:** 2024-09-20

**Authors:** Antonio Vita, Stefano Barlati, Roberto Cavallaro, Armida Mucci, Marco A. Riva, Paola Rocca, Alessandro Rossi, Silvana Galderisi

**Affiliations:** ^1^ Department of Clinical and Experimental Sciences, University of Brescia, Brescia, Italy; ^2^ Department of Mental Health and Addiction Services, Azienda Socio-Sanitaria Territoriale (ASST) Spedali Civili of, Brescia, Italy; ^3^ Department of Clinical Neurosciences, Scientific Institute for Research, Hospitalization and Healthcare (IRCCS) San Raffaele Scientific Institute, Milan, Italy; ^4^ School of Medicine, Vita-Salute San Raffaele University, Milan, Italy; ^5^ Department of Mental and Physical Health and Preventive Medicine, University of Campania Luigi Vanvitelli, Naples, Italy; ^6^ Department of Pharmacological and Biomolecular Sciences, University of Milan, Milan, Italy; ^7^ Biological Psychiatry Unit, Scientific Institute for Research, Hospitalization and Healthcare (IRCCS) Istituto Centro San Giovanni di Dio Fatebenefratelli, Brescia, Italy; ^8^ Department of Neuroscience “Rita Levi Montalcini”, University of Turin, Turin, Italy; ^9^ Department of Biotechnological and Applied Clinical Sciences, Section of Psychiatry, University of L’Aquila, L’Aquila, Italy

**Keywords:** CIAS, cognitive impairment, cognitive remediation, evidence-based, psychosocial functioning, psychosocial rehabilitation, schizophrenia, social cognition

## Abstract

A considerable proportion of patients with schizophrenia perform below population norms on standardized neuropsychological tests, and the performance of those performing within normal range is lower than predicted based on parental education. Cognitive impairment predates the onset of psychosis, is observed during symptom remission and in non-affected first-degree relatives of patients. At the present time, cognitive deficits are regarded as key features of schizophrenia, important determinants of poor psychosocial outcome and targets for both pharmacological and non-pharmacological treatment strategies. A group of eight key opinion leaders reviewed and discussed latest advances in scientific research and current good clinical practices on assessment, management, and treatment of CIAS. In the present paper they summarize the current evidence, identify main gaps between current knowledge and mental health services clinical practice, and provide practical recommendations to reduce the gap.

## 1 Introduction

Cognitive impairment represents one of the core features of schizophrenia and has been considered one of the essential characteristics of the disorder since its earliest conceptualizations (1–5). The presence of a significant cognitive impairment, expressed as a global cognitive performance that is one standard deviation below the population average, can currently be observed in more than 80% of individuals receiving a clinical diagnosis of schizophrenia (6, 7). The frequency and clinical relevance of this issue are such that the international scientific community currently tends to refer to it by using a dedicated term, i.e., Cognitive Impairment Associated with Schizophrenia (CIAS) (8). CIAS involves a wide variety of neurocognitive domains; attention, processing speed, working memory, learning and memory and executive functions are frequently and significantly impaired (1, 9, 10). Social cognition performance also appears to be affected, as significant deficits can be observed in emotion processing, theory of mind, attributional style and social perception domains (11–14).

CIAS is one of the earliest manifestations of the disorder. It can be observed in high-risk subjects that will eventually develop schizophrenia, predates the clinical onset of the disorder, and appears to flare up in severity during the prodromal phase of psychosis (15–17). On the other hand, longitudinal studies attest that the severity of CIAS, in the absence of a dedicated treatment, appears to be substantially stable over the course of life (18–21); however, working memory and social cognition abilities appear to be more affected in chronic phases of the illness, and some patients do face an increase in the severity of CIAS over time, suggesting that a limited but progressive component of deterioration might be present (22–26).

CIAS also involves metacognition, defined as the ability to think about one’s own thoughts (27–29). Unlike neurocognition and social cognition, which are more focused on the level of exactness of perceptions and representations, metacognition focuses on psychological experience synthesis into mental representations with a large variety in terms of complexity, adaptiveness, and flexibility (30). Overall, cognitive impairment in schizophrenia is a consequence of the neurobiological alterations that underlie psychopathological manifestations of the disorder (2, 6). In particular, schizophrenia is characterized by several neurobiological features, which lead to alterations in the neuronal functioning in dopaminergic, cholinergic, glutamatergic, and GABAergic systems, resulting in imbalanced interactions in cortical and subcortical microcircuits (31, 32). Grey matter volume reduction, aberrant neural network organization, altered neuronal functioning and neurotransmission, and excitatory/inhibitory imbalance at a cortical level are all correlates of cognitive dysfunction and appear to represent the neurobiological and pathophysiological bases of cognitive impairment in schizophrenia (3).

The increasing interest for CIAS is mainly due to its impact on the daily life of people diagnosed with schizophrenia. In fact, cognitive performance is strongly related to functional capacity and to psychosocial functioning, and a wealth of high-quality scientific evidence concurs in attesting that CIAS represents one of the core determinants of functional impairment in real life, even more than positive and negative symptoms (33–37). CIAS impact on real-life functioning is attested by both cross sectional studies and longitudinal studies showing that it is a strong predictor of long-term real-life outcomes (33–38). The studies of the Italian Network for Research on Psychoses have provided strong evidence of the impact of CIAS on main aspects of real-life functioning of more than 900 individuals with schizophrenia (34, 36), and of its strong predictive value of real-world functional outcomes at a 4-year follow-up in more than 600 participants (38).

Recently, a group of eight key opinion leaders (KOLs) reviewed and discussed latest advances in scientific research and current good clinical practices on assessment, management and treatment of CIAS. In this paper they report on main gaps between current knowledge and mental health services clinical practice, and provide practical tips to reduce the gap.

## 2 Materials and methods

The present paper is the result of two full-day expert online meetings, in which eight expert psychiatrists (KOLs), representative of different Italian academic and clinical contexts and actively involved in the clinical management of schizophrenia, discussed their expertise on CIAS. The KOLs analyzed the available literature from 2001 to 2024, and discussed the evidence and unmet needs on CIAS. To identify studies exploring the definition, the assessment and treatment of CIAS, searches were conducted on electronic databases PubMed, Scopus, and Google Scholar. Key terms for the searches included “schizophrenia” and “cognition” or “cognitive performance” or “CIAS” or “cognitive impairment” or “social cognition” or “psychosocial functioning” and “cognitive remediation” or “evidence-based intervention” or “psychosocial rehabilitation”.

Both original studies and reviews were considered of interest for the purpose of the present work, as reviews usually also include assessments, perspectives, and comments by the authors and therefore a narrative and critical perspective could provide valuable information beyond the sum of the included studies. References of interest emerging from the citations of included works were also taken into account for inclusion. As the selection of the included literature did not follow a systematic procedure, given the structure and the aims of the present expert opinion paper, it should be noted that the studies present in the review to do not represent the totality of the works investigating the explored topic.

During the first meeting, KOLs were encouraged to share their own perspectives on open issues and future directions regarding research and clinical management of CIAS. In particular, they provided a summary of the state of the art on available assessment instruments, the available psychosocial and pharmacological treatment options and the impact of psychiatric and physical comorbidities affecting cognitive functioning in schizophrenia. An outline of the present paper was discussed and each KOL was assigned the task of reviewing the available evidence on one of the included topics, and integrating the review with her/his own clinical expertise. The manuscript resulting from the different contributions was shared before the second meeting and then discussed during the meeting, when the identified topics were thoroughly discussed, and a group consensus was reached on each topic.

The final version of the manuscript reflects the common view reached by the group of experts in an undivided and genuine manner.

## 3 Assessment of CIAS

The most recent international guidance dedicated to the assessment of CIAS, was ideated and redacted by the Schizophrenia Section of the European Psychiatric Association (39). The European guidance provides a systematic review of the evidence and several recommendations for the assessment of cognitive functions in schizophrenia both in research settings and in real-world clinical practice. In particular, the guidance recommends a separate assessment of the different neurocognitive domains identified by the Measurement and Treatment Research to Improve Cognition in Schizophrenia (MATRICS) initiative (40, 41) and by the Social Cognition Psychometric Evaluation (SCOPE) project (40). This is of particular importance considering that CIAS shows a significant inter-individual heterogeneity in terms of both overall severity and involved domains (42–44).

The MATRICS Consensus Cognitive Battery (MCCB) is currently considered the gold standard instrument for neurocognitive performance assessment (39). The MCCB provides an accurate assessment of the six core neurocognitive domains (attention/vigilance, speed of processing, working memory, verbal learning and memory, visual learning and memory, and executive functions) and incudes also a unidimensional assessment of social cognition performance (9, 45). However, the MCCB is quite lengthy, requiring 60-90 minutes to be completed, and is therefore more suited for research settings, than for everyday clinical practice.

The Brief Assessment of Cognition in Schizophrenia (BACS) battery (46) provides a less comprehensive assessment, as it does not consider all cognitive domains, and has a lower grade of recommendation as compared to the MCCB (39). However, the BACS requires only 35 minutes to be completed, so it may represent a useful instrument in routine clinical settings, especially in contexts with limited resources.

Also the Screen for Cognitive Impairment in Psychiatry (SCIP) (47) provides the assessment of only four of the core MATRICS domains; however, due to its very brief administration time (15 minutes), it shows a potential usefulness as a screening tool in both clinical practice and research settings, particularly for targeted trial recruitment.

Taking into account patients’ perspective and experiences (48, 49), interview-based instruments, such as the Schizophrenia Cognition Rating Scale (50) or the Cognitive Assessment Interview (51–53), requiring 15-30 minutes to complete, should be considered for the assessment of CIAS in clinical settings and as co-primary measures in future studies and trials.

Social cognition represents an independent, multi-domain dimension that should also be thoroughly investigated. According to the SCOPE project studies (12, 54), the tests with the most robust psychometric characteristics are the Bell-Lysaker Emotion Recognition Task (BLERT) (55), for the emotion processing domain, and the Hinting Task (56) and The Awareness of Social Inferences Test (TASIT) (57), for the theory of mind/mentalizing domain. The BLERT and the Hinting Task require only between 6 to 8 minutes to be completed, and, according to the EPA guidance, have a higher grade of recommendation in comparison with the TASIT, which also shows good psychometric qualities but can require up to 45 minutes.

As regards the attributional style and social perception domains, currently available tests do not show satisfying psychometric qualities and are not recommended for research or clinical use. Future efforts in the field should be focused on developing and validating effective measures to appropriately assess CIAS in these two higher-order social cognition domains.

Metacognition can be assessed by a clinician with the Metacognitive Assessment Scale - Abbreviated (MAS-A) (58) after a semi-structured interview, i.e., the Indiana Psychiatric Illness Interview (59). This scale evaluates the comprehension of one’s own and others’ mental states and the ability to work through one’s representations and mental states. The interview and the evaluation last about 45 minutes.

An issue that currently deserves scientific and clinical attention due to its important clinical implications is the timing of the assessment. The long-term consequences of CIAS, particularly if left untreated, and its negative impact on real-life and recovery outcomes (22, 38, 60–62) requires that a treatment-oriented assessment be performed as soon as possible during the clinical course of the disorder. Of course, a single assessment is not sufficient for the entire lifetime of a person diagnosed with schizophrenia. It should be repeated after a cognition-targeting treatment in order to assess response and confirm the effectiveness of the treatment or the need for a different approach or intervention. CIAS evaluation should also be repeated over time, to assess and manage the recovery process of individual patients and, from a research perspective, to identify the factors that contribute to long-term cognitive decline (19). However, the best time to perform these assessments and the optimal time interval between long-term serial assessments have yet to be defined, and more research is currently needed to provide clear recommendations.

The assessment of CIAS in subjects with at risk mental states also represents an issue that warrants further research efforts (15–17). It might represent a valuable screening tool to identify individuals with a higher probability of conversion to full-blown psychotic disorders, and could help to timely identify subjects requiring cognition-targeting treatments. However, the context and the modalities, as well as the practical usefulness and cost-effectiveness, of such an approach remains to be defined. The recommendations and position statements relevant to CIAS assessment are reported in the **Box 1**.

Box 1 **Recommendations and position statements in managing CIAS: focus on assessment.**

- Neurocognitive and social cognition performance should be carefully assessed in patients with schizophrenia, in all phases of the disorder, as well as in CHR subjects, considering the important negative effects of CIAS on functional outcomes and QoL.
- Cognitive performance should be assessed in different stages of the illness, at the start, and at the completion of dedicated treatment programs.
- MCCB represents the most appropriate and complete validated tool currently available to assess CIAS, also in subjects at CHR. BACS could be used as an alternative assessment tool for its short time of administration. SCIP can be used as a screening instrument. Validated Italian versions and normative values are available for all these instruments.
- Interview-based instruments, such as SCoRS and CAI, can be used in the assessment of CIAS in routine clinical context and as co-primary measures in clinical trials. Validated Italian version are available for both measures. Training opportunities are offered by the Department of Mental and Physical Health and Preventive Medicine of the University of Campania L. Vanvitelli; the Department of Neuroscience of the University of Torino; the Department of Clinical Neurosciences, IRCCS San Raffaele Scientific Institute, Milan, Italy; School of Medicine, Vita-Salute San Raffaele University, Milan, Italy; and by the Department of Mental Health and Addiction Services of the University of Brescia.
- Social cognition performance should be assessed with the BLERT and the Hinting task for emotional processing and ToM domains, respectively. TASIT can be used to obtain useful information on both domains. For the latter instrument, there is a validated Italian version with normative reference values, which can be requested from the Department of Mental and Physical Health and Preventive Medicine of the “Vanvitelli” University of Naples. For other social cognitive domains, no available test has sufficiently reliable psychometric properties to justify its recommendation.
- Metacognition should also be considered and assessed with the MAS-A.
- Both self-reports and observer reports of cognitive ability should be taken into account as they give complementary information.
- In the context of limited resources, providing any type of cognitive assessment is better than providing no cognitive assessment at all. The CAI is freely downloadable from the Journal of Psychopathology website (https://old.jpsychopathol.it/article/inter-rater-reliability-and-psychometric-characteristics-of-the-italian-version-of-the-cognitive-assessment-interview-cai/) and can be administered by any professional, psychiatrists, rehabilitation therapists, psychologists with experience in the clinical interviewing of people with schizophrenia.

BACS, Brief Assessment of Cognition in Schizophrenia; BLERT, Bell Lysaker Emotion Recognition Task; CAI, Cognitive Assessment Interview; CHR, clinical high risk; CIAS, Cognitive Impairment Associated with Schizophrenia; MAS-A, Metacognitive Assessment Scale - Abbreviated; MCCB, MATRICS Consensus Cognitive Battery; QoL, Quality of Life; TASIT, The Awareness of Social Inference Test; ToM, Theory of Mind; SCIP, Screen for Cognitive Impairment in Psychiatry; SCoRS, Schizophrenia Cognition Rating Scale.

## 4 Management and treatment of CIAS

CIAS should not be considered a static and unmodifiable feature. Indeed, several therapeutic options are currently available to treat this condition. In fact, the Schizophrenia section of the European Psychiatric Association recently produced a guidance dedicated specifically to the management and treatment of CIAS (63).

### 4.1 Pharmacological mechanisms and CIAS

In addition to providing cognition-oriented interventions or therapies, the improvement of CIAS can result from promoting treatments or interventions that boost cognitive functioning while managing and avoiding factors that contribute to and even worse CIAS. Indeed, while CIAS may be a consequence of specific neurobiological alterations, people living with schizophrenia are also exposed with a considerable frequency and intensity to factors that have a negative impact on cognitive performance (1, 2, 6, 63).

When considering pharmacological treatments, antipsychotic medications represent the cornerstone of the clinical management of schizophrenia, and they are the main strategy to maintain clinical stability, allowing the implementation of rehabilitation programs and interventions and, overall, the process of recovery (2, 61, 64–66). However, different antipsychotics, while showing similar effectiveness on psychotic symptoms, have substantial different effects on cognitive outcomes. Today, the wealth of evidence showing that second-generation antipsychotics are superior to first-generation ones with respect to CIAS is such that an SGA is clearly and strongly recommended, as is the switch to an SGA in people showing CIAS and treated with a first-generation agent (67–70). However, the available evidence supporting the superiority of one drug over the others concerning the impact on CIAS is currently insufficient, and more research is needed (71). Meta-analytic studies exploring this issue in depth have already been planned and are currently undergoing (69).

Within the heterogeneous group of SGAs, several mechanisms can contribute to the potential improvements of CIAS. For example, partial agonism at the D2/D3 receptors could eventually promote cognition, although specific studies are needed to establish to what extent such mechanism may indeed contribute to CIAS improvement (72–74).

One important issue to be borne in mind is that most SGAs interact with several neurotransmitter receptors at therapeutic doses, suggesting that such mechanisms may interact with each other to regulate the functional activity of specific brain circuits, including those that play a relevant role for CIAS. For example, different antipsychotics are associated with an increased release of dopamine in the prefrontal cortex, an effect that can be sustained by specific mechanisms including the antagonism of 5HT7 receptors or the agonism/partial agonism at 5HT1a receptors (75). The new antipsychotic drugs recently introduced in the clinical practice seem to have promising pharmacodynamic profiles likely to improve specific cognitive domains in patients with schizophrenia. In particular, lurasidone, cariprazine, and brexpiprazole have been studied for their potential pro-cognitive effects in schizophrenia patients, demonstrating, although preliminarily, positive effects in several cognitive domains. Some research suggested that lurasidone may have cognitive benefits, including improvements in processing speed, attention, and executive functions in schizophrenia patients (76–79). Other studies on the pro-cognitive effects of the two new dopamine partial agonists, i.e., cariprazine and brexpiprazole, indicated improvements in working memory, learning, and executive abilities (80, 81). These positive effects on cognitive functions contribute to enhancing daily functioning, quality of life, and the overall well-being of persons with schizophrenia, though individual responses to treatment may vary.

On the other end, some pharmacological mechanisms have a negative impact on cognition and may therefore represent an obstacle to functional improvement. For example, the total anticholinergic burden, which is composed mainly by direct anticholinergic medications and the anticholinergic effect of some antipsychotics, is directly related to more severe CIAS (82–84) and lower functional capacity (85, 86), and directly limits the effectiveness of cognition-oriented interventions targeting CIAS (85, 87, 88). Therefore, avoiding the use of anticholinergic medications and of antipsychotics with a substantial anticholinergic component could substantially improve CIAS. More research is currently needed to confirm and quantify the extent of such improvement, and the practical usefulness of such an approach.

Benzodiazepines also have a negative impact on CIAS. They are frequently prescribed to people living with schizophrenia (89), in particular to elderly ones (90). Indeed, benzodiazepines are well-recognized as a risk factor for cognitive decline in elderly subjects (91–93). Limiting the use of benzodiazepines in the treatment of schizophrenia, particularly for prolonged periods, may also have a positive influence on CIAS.

The recommendations and position statements on pharmacological management in CIAS treatment are reported in **Box 2**.

Box 2 **Recommendations and position statements in managing CIAS: focus on pharmacological treatment.**

- SGAs are recommended for their favorable cognitive profile compared to FGAs.
- For patients treated with FGAs, a switch to SGAs should be considered.
- Among SGAs, no clear superiority of one drug over the other ones was found regarding the efficacy on CIAS.
- Avoiding the use of anticholinergic medications and of antipsychotics with a substantial anticholinergic component could substantially improve CIAS.
- Limiting the use of benzodiazepines in the treatment of schizophrenia, particularly for prolonged periods, may have a positive influence on CIAS.

CIAS, Cognitive Impairment Associated with Schizophrenia; FGAs, first-generation antipsychotics; SGAs, second-generation antipsychotics.

### 4.2 Management of factors affecting cognitive functioning

It is important to point out that not all factors showing a negative impact on CIAS are of a pharmacological nature.

Metabolic conditions, in particular metabolic syndrome and its individual components, are a frequent feature in people living with schizophrenia, with important consequences on clinical, health-related and even mortality outcomes (94–97). Metabolic issues have been shown to have a substantial negative impact on cognitive functioning in the general population (98–104), and are also directly related to the severity of CIAS (105–108). People living with schizophrenia show a genetic vulnerability to metabolic conditions (109–112), but several environmental factors such as smoking, inadequate diet and sedentary lifestyle also play a relevant role and can be modified with appropriate education and targeted interventions (113–117). Antipsychotic medications are also considered another important source of metabolic disruption in people living with schizophrenia (118–120); however, antipsychotic molecules show substantial differences as regards their metabolic profile, and optimizing the treatment program also in this regard could provide substantial benefits in the perspective of CIAS treatment (121).

Sleep disturbances represent another frequent feature in people living with schizophrenia (122–124), and the negative impact of sleep disturbances on cognitive outcomes in the general population, as well as the role of sleep disturbances and alterations as a risk factor for cognitive decline in elderly individuals, are well known (125–129). However, the relationship between sleep disturbances and CIAS has been explored only in a limited manner in available scientific literature, and while these two issues appear to be closely connected, recent systematic reviews report that the extent and the implications of their association remain to be more accurately explored (130, 131). In this regard, future studies should explore whether and to what extent treating sleep disturbances and normalizing sleep and circadian rhythm patterns could have a positive impact on CIAS severity.

Finally, alcohol and substance abuse represents another issue that is frequent in people living with schizophrenia and can have a negative impact on CIAS (132–137). In particular, cannabis use has been shown to be associated with negative effects on cognitive performance both for short- and for long-term use (138–140) and can substantially worsen CIAS (141, 142). Similar effects have also been observed for cocaine use (143–145). Beside the improvement of CIAS, effective treatment of substance use disorders in schizophrenia leads to significant long-term improvements in several clinical and health outcomes (146–150), and therefore recognition and treatment of substance use is strongly recommended (64, 151–153).

### 4.3 Cognitive remediation

To date, cognitive remediation (CR) represents the intervention that has demonstrated the highest level of recommendation available in CIAS treatment. CR is a behavioral training-based psychosocial intervention specifically targeting cognitive impairment (154, 155). Several large and recent meta-analyses have shown that CR provides significant CIAS enhancement, both in neurocognition and social cognition, that in turn leads to an improvement in real-world daily functioning (156–159). CR also shows a good overall acceptability profile (160) and it is usually described as a positive experience by participants (161–163). Moreover, some CR interventions show minimal resource requirements and can be easily implemented in everyday real-world psychiatric rehabilitation settings (164, 165), even in contexts with very limited available resources and low-income regions such as Western Sub-Saharan Africa (166).

However, some elements of CR are essential to its effectiveness to the extent that they can be considered core treatment components, and should therefore always be taken into account in the CR implementation process. In particular, the presence of an active and trained therapist, the repeated practice of cognitive exercises, the structured development of cognitive strategies, and use of techniques to improve the transfer of cognitive gains into the real-world have been identified by expert as essential (154), and recent meta-analytic investigations have confirmed their real-world importance (158, 160).

Furthermore, although there are significant strengths regarding the usefulness of integrating CR with supported employment interventions in order to achieve better outcomes, including also getting a job (165),, there are many patients, particularly those with less cognitive deficits, who obtain and sustain a competitive employment, even without an integration with CR, but only with dedicated placement and support interventions (64). In this regard, the importance of an initial assessment that is as precise and comprehensive as possible is reiterated, in the view to accomplish a targeted and tailored rehabilitation intervention.

Genetic characteristics of participants might also influence the response to CR interventions: some studies for instance report that individuals with a particular allelic configuration of a gene related to synaptic COMT efficacy, known as ‘Met carriers’ and characterized by a higher quantity of available synaptic dopamine in prefrontal cortex regions compared to the more diffuse Val/Val allelic presentation, show an overall better response to CR interventions (167, 168). Within this variability, pharmacological treatment also appear to play an important role, and if subjects under clozapine treatment are considered, the aforementioned results appear to be reversed: in fact, Val/Val allele carriers treated with clozapine show better CR response (169). Another gene that potentially influences CR response and CR-pharmacological therapy interactions is the one coding EAAT2: individuals with the T/T genotype, compared to those with the T/G allele, show better overall CR response. However, EAAT2 expression is negatively influenced by clozapine, so this effect was not observed in T/T participants treated with this antipsychotic (170).

The recommendations and position statements on CR interventions in CIAS treatment are reported in the **Box 3**.

Box 3 **Recommendations and position statements in managing CIAS: focus on non-pharmacological interventions.**

- CR is recommended for the treatment of CIAS.
- Social cognitive training is recommended for the treatment of social cognitive deficits in schizophrenia.
- CR interventions should be delivered by a trained therapist and integrated in a psychosocial rehabilitation program.
- In a context of limited resources, a paper and pencil version of the Cognitive Remediation Therapy (CRT) can be obtained at no cost.
- Training opportunities for computerized and paper and pencil CR programs are offered by expert centers that can be contacted through the Italian Group for the study and treatment of Cognition in Psychiatry (Gruppo Italiano per lo studio e il trattamento della Cognitività in Psichiatria, GICoPsi).
- The Good Clinical Practice Recommendations for Adult Psychosocial Rehabilitation, published by the Italian Society of Psychosocial Rehabilitation, strongly recommend CR for people living with schizophrenia. These recommendations have been disseminated through the Regional Sections of the Society and might help promoting a larger implementation of CR in Italian Mental Health Departments.
- Physical exercise should be integrated into rehabilitation projects considering its positive effects on CIAS.

CIAS, Cognitive Impairment Associated with Schizophrenia; CR, cognitive remediation.

### 4.4 Physical exercise

Another treatment that has shown substantial positive effects on CIAS is physical exercise. Although the amount of scientific evidence attesting the effectiveness of physical exercise in improving CIAS is not as extensive as that available for CR, several meta-analyses converge in showing reliable positive effects (171–173). A recent, large meta-analytic study investigated the role of moderators of effect, confirming that the most effective form of physical exercise to treat CIAS is aerobic exercise; it also reported a superior effect of group exercise, with the supervision of trained professionals, and with a dose-dependent effect, starting from a duration of ≥90 min per week for ≥ 12 weeks (174). These are actually very significant findings, as the lack of knowledge on moderators of effects limited the possibility of providing clear instructions on how to implement physical exercise-based intervention in clinical practice, and therefore limited the recommendation of physical exercise in CIAS guidelines (63). However, in light of these findings, it is possible to fully consider physical exercise as an effective evidence-based intervention for CIAS, which can also provide substantial metabolic and global health benefits (64, 117, 175). Moreover, physical exercise-based interventions can be easily and practically combined with CR interventions into structured rehabilitation programs that associate and increase the effectiveness of both treatments on CIAS (176–178).

The recommendations and position statements on physical exercise in CIAS treatment are reported in the **Box 3**.

### 4.5 Non-invasive brain stimulation

Non-invasive brain stimulation techniques, such as repetitive transcranial magnetic stimulation (rTMS) and transcranial direct current stimulation (tDCS), represent treatments that are gaining increasing attention in the scientific community, with some preliminary results suggesting benefits also in the treatment of CIAS (179–182). However, some meta-analytic studies also report negative results (183, 184): given these conflicting findings and the scarcity of available studies, it is not currently possible to draw any definitive conclusion on the effectiveness of non-invasive brain stimulation in CIAS. In this regard, more research, and in particular well-conducted, independent randomized controlled trials are currently needed to obtain more data. These future studies should also focus on comparing different treatment modalities (different electrode positioning, different intensity of stimulation, and different duration), in order to better identify and codify effective treatment programs.

The recommendations and position statements on non-invasive brain stimulation techniques in CIAS treatment are reported in the **Box 4**.

Box 4 **Recommendations and position statements in managing CIAS: focus on non-invasive brain stimulation techniques.**

- NIBS techniques, such as rTMS and tDCS, represent a treatment that is gaining increasing attention in the scientific community, and some preliminary findings suggest that they could provide benefits in the treatment of CIAS.
- The available literature does not allow to recommend rTMS as an evidence-based intervention for CIAS.
- Some encouraging findings suggest that tDCS could provide some positive cognitive effects, but it cannot be currently recommended as an evidence-based intervention for CIAS.
- NIBS are not currently recommended as a standalone treatment to be used in clinical practice in the treatment of CIAS, but they should be considered as an add-on therapy in an integrated perspective.

CIAS, Cognitive Impairment Associated with Schizophrenia; NIBS, non-invasive brain stimulation; rTMS, *Repetitive* Transcranial Magnetic Stimulation; tDCS, *Transcranial* Direct Current Stimulation.

### 4.6 Novel pharmacological strategies for CIAS

Pharmacological treatment of CIAS is a field of research that is gaining increasing scientific attention and that is showing some promising findings. In fact, while the available evidence is not sufficient to recommend any specific medication as an evidence-based treatment for CIAS, several potentially beneficial molecules are currently being developed and investigated. Preliminary, but promising results have been observed in particular for molecules targeting glutamatergic pathways, primarily through the modulation of glutamate N-methyl-d-aspartate (NMDA) receptors (185, 186). For instance, N-acetylcysteine interacts with the redox/glutathione sensitive site of the NMDA receptors, showing potential neuroprotective effects (187, 188). Glycine transporter (GlyT1) inhibitors are another class of molecules that increase the synaptic levels of glycine, a co-agonist at NMDA receptors, and may therefore potentiate glutamatergic function. Indeed, very promising results have been observed with the GlyT1 inhibitor iclepertin (73, 189). An alternative strategy is represented by luvadaxistat, a D-amino acid oxidase inhibitor that increases the levels of D-serine, which is also a co-agonist at glutamate NMDA receptors. Luvadaxistat has shown substantial effectiveness in murine models (188, 190) and promising results in human trials (191). D-serine (192) and memantine (193) have also been investigated as potential candidates for CIAS treatment.

One interesting novel drug is KarXT, a combination of the M1/M4 agonist Xanomeline with the peripheral antimuscarinic agent trospium, which was effective in a phase III study in reducing positive and negative symptoms with some indication also for an improvement of CIAS (194–197).

Another compound under development for the treatment CIAS is RL-007, which is targeting the cholinergic, NMDA and GABA type B receptor systems.

Anti-inflammatory and immunomodulator molecules represent another very important class of medications that could provide considerable benefits in the treatment of schizophrenia and of CIAS in particular (198, 199).

Other agents such as intranasal oxytocin are currently being investigated as specific treatments for social cognition deficits (200, 201).

### 4.7 Real-life implementation of CIAS management

The impact of CIAS on quality of life and real-life functioning in schizophrenia has promoted the development of international guidance papers and guidelines on CIAS assessment and treatment, with a strong recommendation to offer CR to all subjects with schizophrenia, in all the phases of the disorder (39, 63, 202–204). In Italy, the Italian Society for Psychosocial Rehabilitation published recommendations of good clinical practice (205), with the same strong advice to offer CR to people living with schizophrenia. Both the European guidance paper and the Italian recommendations suggest reducing the negative impact on CIAS of anticholinergic or benzodiazepine medications, as well as of extrapyramidal side effects, particularly frequent with first generation APDs (39, 63, 205). The relevant recommendations and position statements are reported in the **Box 2**.

The translation into clinical practice of the recommendations concerning CR is still limited both in high-income and poor/middle income countries (206–208). Main barriers are shortage of qualified personnel and economic resources (206–208). The recommendations and suggestions for practical implementation of CR programs in Italy, also in the context of regionally underfunded mental health care, are provided in the **Box 3**.

## 5 Discussion

CIAS represents an essential feature of schizophrenia, and a wealth of scientific literature provides substantial guidance on how to assess and treat this domain both in a research perspective and in real-world clinical practice. However, several important questions regarding CIAS currently remain unanswered, and several relevant issues remain to be addressed.

One particularly problematic and currently open issue regards the implementation of CIAS assessment and of evidence-based psychosocial interventions in real-world clinical settings. In fact, even in high income countries and in contexts with a high amount of available resources, their adoption in rehabilitation settings and in mental health services remains uneven and partial, although the available literature is clearly consistent in attesting the usefulness of these practices and the effectiveness of these interventions, and available guidelines clearly recommend their use and also provide suggestions for their practical implementation (209, 210). Therefore, on the one hand more research is currently needed to better identify barriers and facilitators to the implementation of evidence-based interventions, including CIAS assessment and treatment, in clinical practice; on the other hand, more efforts are needed by researchers to disseminate information regarding CIAS assessment and treatment and more attention should be dedicated by both academics and by policy makers to close, or at least reduce, the “bench-to-bedside” gap regarding CIAS.

More evidence on neurobiological bases of CIAS could provide substantial insight on how to maximize the effectiveness of the available treatments: genetic and biological markers are correlated to different effects of CR and play a role in CR-antipsychotic interactions on CIAS effects (168–170), and more knowledge in this field could help in conceiving and in developing new personalized treatment plans targeting CIAS (2). In this perspective, better understanding the relationship between CIAS and other core symptoms dimensions of schizophrenia, such as psychotic symptoms, primary and secondary negative symptoms (211–215), and autistic symptoms (216–221), which are all deeply inter-related and interconnected (2, 34, 36, 37), could provide valuable insight in the characterization of individual patients and in development and implementation of tailored treatment programs. Negative symptoms are closely related to cognitive impairment, particularly to social cognition (37). It is reported that patients with schizophrenia and deficit syndrome showed a substantially worse cognitive performance, compared to those without deficit syndrome, highlighting the deep connection between negative symptoms and cognitive impairment. More specifically, all the following cognitive domains were worse in subjects with deficit schizophrenia: global cognition, verbal fluency, verbal and visual memory, attention, processing speed, executive functions, and social cognition (11, 212, 213). Positive symptoms are considered a possible cause of secondary negative symptoms (212, 213). Positive symptoms in themselves have been investigated as a potential source of direct neurotoxicity, leading to cognitive impairment, and specific positive symptoms, i.e. delusions, have been associated with specific cognitive impairment, particularly executive dysfunction (30). Autistic symptoms also represent a frequent feature in people with schizophrenia (219). Considered a core characteristic of schizophrenia since its earliest conceptualizations. they have recently increasing scientific attention (32, 216). In fact, autistic symptoms in people living with schizophrenia are associated with overall worse cognitive performance, particularly in social cognitive domains such as Emotion Recognition and Theory of Mind (32, 218, 221). Overall, positive, negative and autistic symptoms and cognitive impairment, albeit clearly distinct in a phenomenological context, are deeply inter-correlated and interconnected (2, 34, 36). Although disentangling this complex network of inter-relationships is beyond the scope of the present work, it is possible to say that the appropriate management of schizophrenia in the real-world clinical practice should directly and carefully manage all these dimensions in concert, as suggested and highlighted in available guidelines (63). Moreover, the potential response to treatment of these dimensions, in particular of negative and autistic symptoms, in people with schizophrenia remain to be thoroughly investigated: in this regard, whether negative and autistic symptoms are susceptible of improvement and if this improvement could be translated into an increase of neurocognitive and social cognitive performance should represent the focus of future dedicated research.

The relationship between metabolic issues and CIAS (105–108) and between sleep alterations and CIAS (130, 131) also requires more scientific attention: in particular, it would be of great scientific and clinical interest to observe and measure whether an optimal treatment of these medical issues could translate into substantial cognitive gains in people with schizophrenia and change the overall severity and trajectory of CIAS.

CIAS and aggressive behavior also appear to share a connection (222–225): better investigating this relationship, exploring whether an appropriate treatment of CIAS might have a positive impact on outcomes, such as the risk of aggressive behavior and violent acts, represents an interesting research direction.

However, to explore all the aforementioned interconnections, in particular to provide data that can be combined and examined across studies, CIAS assessment should performed by researchers in a homogenous manner, avoiding heterogeneity that could complicate or completely hinder the synthesis of result. In this regard, attaining to available international guidance could be an essential arrangement (39).

Finally, one issue that requires greater attention is intrinsically related to available CIAS treatments: even the most effective available treatments, such as CR and psychical exercise, only appear to provide small-to-moderate sized effects; the size of effects fully increases to the moderate range if core treatment ingredients are implemented and if moderators of effect are taken into account (158, 174), but no available intervention can currently provide moderate-to-large effects which could radically and rapidly change the real-world experience of people living with schizophrenia. The same holds true for available experimental pharmacological agents (63, 186). Therefore, the scientific community, including clinical researchers, as well as neurobiology experts, should focus on developing new treatment approaches for CIAS, exploring also the opportunities presented by novel biological and technological developments, such as brain organoids (226–228), gene-editing techniques (229–231), and artificial intelligence algorithms (232–234).

We believe that the present opinion expert paper could have several clinical implications for mental health services, in particular regarding the current evidence on good clinical practices, on the assessment, on the management, and on the treatment of CIAS, also taking into account their feasibility in a real-world clinical setting. Overall, the manuscript summarizes the current scientific evidence concerning CIAS, identifying the main gaps between the current knowledge and the mental health services clinical practice, and provides practical recommendations to reduce them, with the ultimate goal to improve patients outcomes, in order to achieve recovery.

In conclusion, CIAS is a domain of essential importance in schizophrenia, and the available literature provides substantial insight on its assessment and treatment both in research settings and in clinical practice. However, a significant bench-to-bedside gap is currently present in both the assessment and the treatment of CIAS, and more effort should be placed in order to address this issue at different levels. Moreover, several relevant questions regarding CIAS currently remain unanswered, and future research should focus on providing findings that could have a valuable impact on the treatment of CIAS and, consequently, on the real-world life and on the recovery process of people living with schizophrenia.
